# Perioperative immunotherapy: the main clinical treatment for resectable non‐small cell lung cancer

**DOI:** 10.1002/mco2.498

**Published:** 2024-02-28

**Authors:** Zhijun Yuan, Mengyuan Yang, Xian Zhong

**Affiliations:** ^1^ Department of Radiation Oncology (Key Laboratory of Cancer Prevention and Intervention, China National Ministry of Education, Key Laboratory of Molecular Biology in Medical Sciences, Zhejiang Province, China), The Second Affiliated Hospital Zhejiang University School of Medicine Hangzhou Zhejiang China; ^2^ Department of Medical Oncology, The Second Affiliated Hospital Zhejiang University School of Medicine Hangzhou Zhejiang China; ^3^ Zhejiang Provincial Clinical Research Center for CANCER Hangzhou Zhejiang China

## Abstract

Phase 3 clinical trials of perioperative immunotherapy for resectable non‐small cell lung cancer (NSCLC): In recent years, immunotherapy for NSCLC is not only limited to advanced disease, but also has shown gratifying efficacy for early resectable NSCLC. With the publication of the results of several phase 3 clinical trials, perioperative immunotherapy will become one of the main treatment modalities for resectable NSCLC.

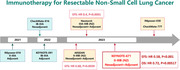

1

The results of the first interim analysis of KEYNOTE‐671 (NCT03425643), led by Wakelee et al.,^1^ were recently presented orally at the American Society of Clinical Oncology (ASCO) 2023 meeting and this breakthrough was also published in *The New England Journal of Medicine*, at the same day. It is mainly emphasized that perioperative treatment of pembrolizumab (immune checkpoint inhibitor; ICI) in resectable non‐small cell lung cancer (NSCLC) can significantly prolong event‐free survival (EFS), but also significantly improve major pathological response (MPR) and pathologically complete response (pCR).

For resectable NSCLC, a large proportion of patients still experience disease recurrence after radical resection. However, even with active adjuvant or neoadjuvant chemotherapy, the improvement in 5‐year overall survival (OS) rate was also only about 5%. Thus, there is an urgent need for a more effective treatment for those patients. Immunotherapy, as a powerful treatment that has developed rapidly in recent years, has not only shown good efficacy in advanced lung cancer, but also has repeatedly shown promising results for resectable NSCLC.^2^ Currently, nivolumab (CheckMate‐816, NCT02998528) and atezolizumab (IMpower‐010, NCT02486718)/pembrolizumab (KEYNOTE‐091, NCT02504372) have received the United States Food and Drug Administration approval for their use in neoadjuvant therapy and adjuvant therapy, respectively. With only the above single approaches, there is still a certain risk of recurrence and eventual death from NSCLC. Therefore, it is worth looking forward to whether the perioperative immunotherapy mode adopted by KEYNOTE‐671 can effectively improve the survival status of resectable NSCLC.

KEYNOTE‐671 is a double‐blind, randomized placebo‐controlled phase 3 study evaluating the perioperative treatment of pembrolizumab in resectable NSCLC. The trial totally enrolled 797 untreated stage II, IIIA, or IIIB (N2 node stage) NSCLC patients, who were randomly assigned to receive pembrolizumab or placebo in a 1:1 ratio. The treatment mode used were pembrolizumab or placebo combined with cisplatin‐based neoadjuvant chemotherapy (for up to 4 cycles) + surgery + adjuvant therapy with pembrolizumab or placebo (for up to 13 cycles). The primary endpoints were EFS (the time from randomization to the first occurrence of local progression that precluded the planned surgery, unresectable tumor, progression or recurrence, or death) and OS, and the secondary endpoints were MPR and pCR.

The median follow‐up for this interim analysis was 25.2 months (range 7.5–50.6). The data showed that pembrolizumab was superior to placebo on most endpoints. Perioperative pembrolizumab treatment reduced the risk of disease recurrence, progression, and death in patients with resectable NSCLC by 42% (hazard ratio: 0.58; 95% confidence interval [CI], 0.46–0.72; *p* < 0.001), median EFS were not reached (95% CI, 34.1 months to not reached) and 17.0 months (95% CI, 14.3–22.0) in the pembrolizumab and placebo groups, respectively. The predicted 24‐month EFS rates were 62.4% (95% CI, 56.8–67.5) and 40.6% (95% CI, 34.8–46.3), respectively. In the subgroup analysis, the EFS benefit with pembrolizumab was generally consistent across all subgroups examined. Among them, patients with current smoker, stage III, high PD‐L1 expression, MPR, or pCR after neoadjuvant therapy could obtain better EFS. It is worth suggesting that EFS was beneficial in the pembrolizumab group, regardless of PD‐L1 expression and MPR or pCR after neoadjuvant therapy. Both secondary endpoints showed statistically significant differences between the two comparison groups (*p* < 0.0001), compared with 11.0% of MPR and 4.0% of pCR in the placebo group, 30.2 and 18.1% in the pembrolizumab group, respectively. Based on this efficacy, ≥grade 3 treatment‐related adverse events occurred in 44.9 and 37.3% of participants in the pembrolizumab and placebo groups, and grade 5 treatment‐related adverse events occurred in 1.0 and 0.8% of participants, respectively, throughout treatment.

In the assessment of clinical efficacy, OS is the gold standard for evaluating clinical benefit of cancer. Based on previous observational studies, improvements in EFS, pCR, and MPR can be expected to prolong OS. However, OS typically requires large samples and longer follow‐up, and can be affected by crossover and follow‐up treatments. KEYNOTE‐671 is the only study that has OS as its primary endpoint among the many studies researching perioperative immunotherapy for resectable NSCLC. Through this interim analysis, the pembrolizumab group has demonstrated a favorable OS improvement trend (HR: 0.73; 95% CI, 0.54–0.99; *p* = 0.02124). In fact, the answer did not keep us waiting long. At the European Society for Medical Oncology Congress that same year, it was made clear that perioperative immunotherapy with pembrolizumab, on the basis of traditional neoadjuvant chemotherapy, could significantly improve OS in patients with stage II, IIIA, or IIIB (N2 node stage) NSCLC (HR: 0.72; 95% CI, 0.56–0.93; *p* = 0.00517).^3^

All the phase 3 clinical trials of perioperative immunotherapy represented by KEYNOTE‐671 only conducted the comparison of ICI and placebo in perioperative period. The role of neoadjuvant immunotherapy or adjuvant immunotherapy alone could not be further explored in a single study. The disease‐free survival (DFS) benefit of adjuvant immunotherapy has been demonstrated in IMpower‐010 and KEYNOTE‐091. Of current interest is whether patients whose surgical pathologic assessment is pCR following neoadjuvant therapy may continue to benefit from adjuvant immunotherapy. Through the subgroup exploratory analysis of KEYNOTE‐671, reductions in EFS events in the pembrolizumab group were still observed in those who received pCR response (HR: 0.33; 95%CI, 0.09–1.22). Therefore, it seems that there is a possibility of further benefit from adjuvant immunotherapy after pCR. At the same time, patients who did not achieve pCR also saw a corresponding reduction in EFS events, and such patients were more in need of adjuvant immunotherapy.

In April, results from AEGEAN (NCT03800134)^4^ and Neotorch (NCT04158440)^5^ were presented at the American Association for Cancer Research (AACR) Annual Meeting 2023 and the ASCO Plenary Series, respectively. Combined with the results of the three phase 3 trials, the perioperative immunotherapy model achieved uniform clinical efficacy (see Table 1 for details). At the same time, no significant increase in treatment‐related adverse events was observed in the combined use of ICI during perioperative period, which was in line with expectations. Significant increases in pCR and MPR were generally seen after neoadjuvant therapy, along with higher planned surgical completion rates (KEYNOTE‐671: 82.1% in pembrolizumab versus 73.2% in placebo) and R0 resection rates (KEYNOTE‐671: 92.0% in pembrolizumab versus 84.2% in placebo).

**TABLE 1:** Phase 3 clinical trials of perioperative immunotherapy for resectable non‐small cell lung cancer.

	Treatment mode	Groups	Median EFS/DFS (month)		MPR (%)		pCR (%)	
IMpower‐010	Surgery + (adjuvant chemotherapy) 16 cycles of adjuvant immunotherapy	Atezolizumab	42.3 (95% CI, 36.0–NR)	HR: 0.79 (95% CI, 0.64–0.96, *p* = 0.02)	–		–	
		Placebo	35.3 (95% CI, 30.4–46.4)					
KEYNOTE‐091	Surgery + (adjuvant chemotherapy) 18 cycles of adjuvant immunotherapy	Pembrolizumab	53.6 (95% CI, 39.2–NR)	HR: 0.76 (95% CI, 0.63–0.91, *p* = 0.0014)	–		–	
		Placebo	42.0 (95% CI, 31.3–NR)					
CheckMate‐816	3 cycles of neoadjuvant therapy^a^ + surgery	Nivolumab	31.6 (95% CI, 30.2–NR)	HR: 0.63 (97.38% CI, 0.43–0.91, *p* = 0.005)	36.9	Difference: 27.9% (95% CI, 19.5–35.9)	24.0	Difference: 21.8% (95% CI, 15.2–28.7)
		Placebo	20.8 (95% CI, 14.0–26.7)		8.9		2.2	
KEYNOTE‐671^1^	4 cycles of neoadjuvant therapy^a^ + surgery + 13 cycles of maintenance therapy	Pembrolizumab	NR (95% CI, 34.1–NR)	HR: 0.58 (95% CI, 0.46–0.72, *p* < 0.001)	30.2	Difference: 19.2% (95% CI, 13.9–24.7, *p* < 0.0001)	18.1	Difference: 14.2% (95% CI, 10.1–18.7, *p* < 0.0001)
		Placebo	17.0 (95% CI, 14.3–22.0)		11.0		4.0	
AEGEAN^4^	4 cycles of neoadjuvant therapy^a^ + surgery + 12 cycles of maintenance therapy	Durvalumab	NR (95% CI, 31.9–NR)	HR: 0.68 (95% CI, 0.53–0.88, *p* = 0.0039)	33.3	Difference: 21.0% (95% CI, 15.1–26.9, *p* = 0.000002)	17.2	Difference: 13.0% (95% CI, 8.7–17.6, *p* = 0.000036)
		Placebo	25.9 (95% CI, 18.9–NR)		12.3		4.3	
Neotorch^5^	3 cycles of neoadjuvant therapy^a^ + surgery + 1 cycle adjuvant therapy^a^ + 13 cycles of maintenance therapy	Toripalimab	NR (95% CI, 24.4–NR)	HR: 0.40 (95% CI, 0.277–0.565, *p* < 0.0001)	48.5	Difference: 40.2% (95% CI, 32.2–48.1, *p* < 0.0001)	24.8	Difference: 23.7% (95% CI, 17.6–29.8, *p* < 0.0001)
		Placebo	15.1 (95% CI, 10.6–21.9)		8.4		1.0	

Abbreviations: CI, confidence interval; DFS, disease‐free survival; EFS, event‐free survival; HR, hazard ratio; MPR, major pathological response; NR, not reached; OS, overall survival; pCR, pathologically complete response.

^a^ Combined platinum‐based chemotherapy.

In summary, the perioperative immunotherapy mode represented by KEYNOTE‐671 has made a historic breakthrough in the treatment of resectable NSCLC, which is supported by a number of research results. However, there are still many topics to be explored, such as postoperative immunotherapy maintenance time, perioperative chemotherapy cycles, and perioperative targeted therapy for people with driver gene mutations. Here, we see that better clinical outcomes can be achieved by combining ICI without adding additional safety costs. This treatment model may become one of the main treatment models for resectable NSCLC in the near future.

## AUTHOR CONTRIBUTIONS

Zhijun Yuan and Mengyuan Yang conceived the study, collected the literatures, and drafted the manuscript. Corresponding author, Xian Zhong, provided corrective comments and revised the manuscript. All authors collaborated to write the article. All authors approved this manuscript for publication.

## CONFLICT OF INTEREST STATEMENT

The authors declare no competing interests.

## ETHICS STATEMENT

Not applicable.

## Data Availability

The data included in this study are available upon request from the corresponding authors.

## References

[1] Wakelee H , Liberman M , Kato T , et al. Perioperative pembrolizumab for early‐stage non‐small‐cell lung cancer. N Engl J Med. 2023;389(6):491‐503. 37272513 10.1056/NEJMoa2302983 PMC11074923

[2] Saw S , Ong BH , Chua K , Takano A , Tan D . Revisiting neoadjuvant therapy in non‐small‐cell lung cancer. Lancet Oncol. 2021;22(11):e501‐e516. 34735819 10.1016/S1470-2045(21)00383-1

[3] Spicer JD , Gao S , Liberman M , et al. LBA56 overall survival in the KEYNOTE‐671 study of perioperative pembrolizumab for early‐stage non‐small‐cell lung cancer (NSCLC). Ann Oncol. 2023(2):S1297‐S1298. 10.1016/j.annonc.2026.08.005 42628840

[4] Heymach JV , Harpole D , Mitsudomi T , et al. Perioperative durvalumab for resectable non‐small‐cell lung cancer. N Engl J Med. 2023;389(18):1672‐1684. 37870974 10.1056/NEJMoa2304875

[5] Lu S , Wu L , Zhang W , et al. Perioperative toripalimab plus chemotherapy for patients with resectable non‐small cell lung cancer: the neotorch randomized clinical trial. JAMA. 2024;331(3):201‐211. 38227033 10.1001/jama.2023.24735 PMC10792477

